# Target identification of natural medicine with chemical proteomics approach: probe synthesis, target fishing and protein identification

**DOI:** 10.1038/s41392-020-0186-y

**Published:** 2020-05-21

**Authors:** Xiao Chen, Yutong Wang, Nan Ma, Jing Tian, Yurou Shao, Bo Zhu, Yin Kwan Wong, Zhen Liang, Chang Zou, Jigang Wang

**Affiliations:** 1 School of Medicine & Holistic Integrative Medicine, and College of Pharmacy, Nanjing University of Chinese Medicine, Nanjing, 210023 China; 2 School of Biopharmacy, China Pharmaceutical University, Nanjing, 210009 China; 3 Artemisinin Research Center, and Institute of Chinese Materia Medica, China Academy of Chinese Medical Sciences, Beijing, 100700 China; 4 The First Affiliated Hospital of Southern University of Science and Technology, The Second Clinical Medical College of Jinan University, Shenzhen People’s Hospital, Shenzhen, 518020 China; 5 Department of Toxicology, School of Public Health, Guangxi Medical University, Nanning, 530021 China

**Keywords:** Target identification, Target identification

## Abstract

Natural products are an important source of new drugs for the treatment of various diseases. However, developing natural product-based new medicines through random moiety modification is a lengthy and costly process, due in part to the difficulties associated with comprehensively understanding the mechanism of action and the side effects. Identifying the protein targets of natural products is an effective strategy, but most medicines interact with multiple protein targets, which complicate this process. In recent years, an increasing number of researchers have begun to screen the target proteins of natural products with chemical proteomics approaches, which can provide a more comprehensive array of the protein targets of active small molecules in an unbiased manner. Typically, chemical proteomics experiments for target identification consist of two key steps: (1) chemical probe design and synthesis and (2) target fishing and identification. In recent decades, five different types of chemical proteomic probes and their respective target fishing methods have been developed to screen targets of molecules with different structures, and a variety of protein identification approaches have been invented. Presently, we will classify these chemical proteomics approaches, the application scopes and characteristics of the different types of chemical probes, the different protein identification methods, and the advantages and disadvantages of these strategies.

## Introduction

Over the last 30 years, natural products have become an important source of new drugs to target various diseases.^1,2^ In contrast to chemically synthesized drugs, drugs derived from natural products possess remarkable advantages in terms of structural novelty, biocompatibility and functional diversity, stemming from long-term natural selection-based optimizations in their evolution.^3^ Statistically, among marked drugs approved by the Food and Drug Administration (FDA) from 1939 to 2016, more than 50% are derived from natural products,^4^ and these compounds are commonly known as natural medicines. For example, elliptinium, a naturally occurring plant alkaloid, has been developed into the anticancer natural medicine Celiptium. It is widely used in multiple cancer therapies, such as breast cancer and renal cell carcinoma.^5,6^ Retapamulin, another natural medicine derived from pleuromutilin produced by *Pleurotus mutilus*, an edible mushroom, is the first in a new class of antibacterial drugs known as pleuromutilins to be approved for use in humans.^7^ In addition to the natural products that have been developed as commercial medications for humans, a number of them are on their way to be patented medicine, such as resveratrol,^8–10^ curcumin,^11–13^ oridonin,^14,15^ etc.^16–18^ However, developing a natural product-based new medicine from random moiety modification is a lengthy and costly process, due in part to the difficulties associated with comprehensively understanding their mechanism of action (MOA) as well as side effects.^19,20^

Interactions with intracellular protein targets are the foundation through which natural products exert their pharmacological activity. Therefore, target identification is the initial key step for the discovery and development of new natural medicines,^21,22^ as this allows the determination of the MOA and side effects. However, further studies on drug and target interaction mechanisms showed that most drugs interact with multiple protein targets rather than a single target.^23–25^ This multitargeted interaction mode makes identifying the true targets of the natural products being investigated substantially more difficult. Therefore, a target identification method that can comprehensively reveal multiple targets of natural products is urgently needed. Several systematic and nonbiased methods for identifying the targets of natural products, such as transcriptome-wide compound signature profiling, chemical genomics approaches and yeast two-hybrid methods, have been developed in recent decades.^26–29^ However, these strategies have disadvantages such as narrow applicability and multiple interference.^30^ With the advancement of molecular biology and the advent of the postgenomic era, an emerging and broadly applicable approach termed chemical proteomics was developed for target identification at the proteomic level.^31,32^

As an important branch of proteomics, chemical proteomics integrates diverse approaches in synthetic chemistry, cellular biology and mass spectrometry.^33^ It is an approach to comprehensively fish and identify multiple protein targets of active small molecules, and it consists of two key steps: (1) probe design and synthesis and (2) target fishing and protein identification. In recent decades, five different types of probes and their respective target fishing methods with different scopes and characteristics for chemical proteomics approaches have been developed^34^ to screen targets for small molecules with different structures. After target fishing, there are also multiple protein identification methods that are suitable for different situations.^35–37^ Numerous pharmacological studies have applied chemical proteomics to identify drug targets and study their MOA,^38–40^ especially in the last few years.^41^ Hence, these studies provides us with a unique background to summarize the recent achievements in this field.

In the present review, we first briefly introduce the chemical proteomics approaches, including their classification and workflow, as well as their advantages and disadvantages. Second, as the initial step of target identification, we provide a glimpse of the synthetic processes of five different types of probes and describe in detail the probes’ application scope and characteristics, as well as their subsequent target enrichment schemes. In the third section of the review, different protein identification methods with distinct scopes, including gel separation and band identification, quantitative proteomics approach, and protein microarray, are described. In the last section, we provide some comments on the future direction of chemical proteomics for the target identification of natural products.

## Chemical proteomics in target identification

**Fig. 1:**
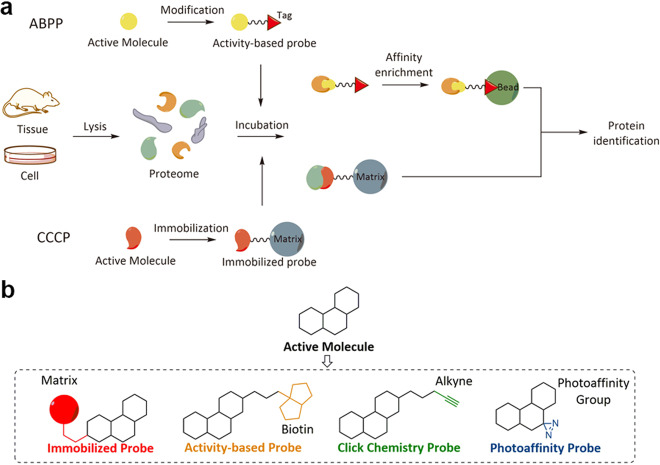
**a** Comparison of activity-based probe profiling and compound-centric chemical proteomics. **b** General molecular structures of different types of chemical proteomics probes

Chemical proteomics is a postgenomic version of classical drug affinity chromatography that is coupled to subsequent high-resolution MS and bioinformatic analyses.^42^ As illustrated in Fig. 1a, chemical proteomics approaches can be divided into two categories according to their different workflows, namely, activity-based protein profiling (ABPP) and compound-centric chemical proteomics (CCCP).^23^

ABPP is a technology that combines activity-based probe and proteomics technologies to identify protein targets of small bioactive molecules to help elucidate their MOA and side effects.^31,43^ In a typical ABPP experiment^44^ (Fig. 1a), probes derived from the parent molecules are first designed and synthesized based on a structure–activity relationship (SAR) study of the parent molecules. The probes should be synthesized as follows: (i) the probes should retain the pharmacological activity of their parent molecules to ensure the accuracy of subsequent target identification; (ii) the probes should allow for easy enrichment of bound protein targets. Next, the probes are incubated with effector living cells, lysate or tissue homogenates, allowing them to completely bind their target proteins. After enrichment with chemical and biochemical techniques, the protein targets are identified through proteomics approaches. The final step is to validate the target information via SPR, MST, ITC, etc. and the corresponding pharmacological effects with the appropriate biological function assays.

CCCP originates from classic drug affinity chromatography, which has been in use for decades.^45–48^ Along with the development of proteomics techniques, CCCP merges the classic method with modern proteomics to identify protein targets of small bioactive molecules at the proteome level. Unlike ABPP (Fig. 1a), the first step of CCCP is to immobilize the drug molecule on a matrix,^23^ such as magnetic or agarose beads. The probe synthesis and immobilization processes will be described in detail in the next section. Similar to ABPP, immobilization should not influence the pharmacological activity of the drug of interest. Subsequently, lysates from cells or tissues are incubated with the affinity matrix, followed by extensive washing to remove nonspecific binders. After complete elution, the enriched proteins are identified with proteomics approaches, and the target information and corresponding pharmacological effects must also be confirmed.

As mentioned above, the chemical proteomics approach may possess many advantages, such as being unbiased and allowing high-throughput at the proteome level, but it also has limitations. With chemical proteomics, the mass of nonspecifically bound proteins and the active metabolites, in addition to the true target proteins, may also be identified, leading to potential false-positive results. Furthermore, proteins that are insoluble in the buffers (e.g., PBS, Tris-HCl) used during the target enrichment process may pass unnoticed through the matrix. Comparing the two chemical proteomics approaches, in contrast to ABPP, the activation state of the identified proteins cannot be detected with CCCP, but CCCP is a more unbiased approach, allowing it to even identify targets with no enzymatic function, thereby facilitating the discovery of novel targets.

## Probe design and synthesis

Designing and synthesizing the probe is the initial and pivotal step for target identification in chemical proteomics approaches. Generally, a probe consists of three parts, which are responsible for its respective functions: (i) a reactive group, which is derived from the parent drug molecule and ensures that it retains its pharmacological activity and ability to bind or modify protein targets; (ii) a reporter tag, such as biotin, an alkyne or a fluorescence group, for target enrichment or detection; (iii) a linker, sometimes cleavable, to connect the reactive group and the reporter tag, and it should be long enough to avoid steric hindrance.^49,50^ However, the structure of the probe may not always be constant. For example, in different chemical proteomics strategies for target identification, the probe might have one or even two of the components omitted. In this section, we will describe the diverse types of probes applied in chemical proteomics target profiling, as well as their design and synthesis, characteristics and scope of application.

### Immobilized probe

In earlier studies, bioactive natural products were covalently immobilized on biocompatible inert resins, such as agarose and magnetic beads, to serve as bait for fish for target proteins in the active proteome (Fig. 1b). Due to the intrinsic properties of the beads, such as their macroscopic size and magnetism, the probe-fished proteins can be easily enriched, which is convenient for subsequent target identification. In the structure of bioactive natural products, various groups, such as sulfhydryl, amino and carboxyl groups, can be utilized for attachment to different active resins, which are commercially available.

**Fig. 2:**
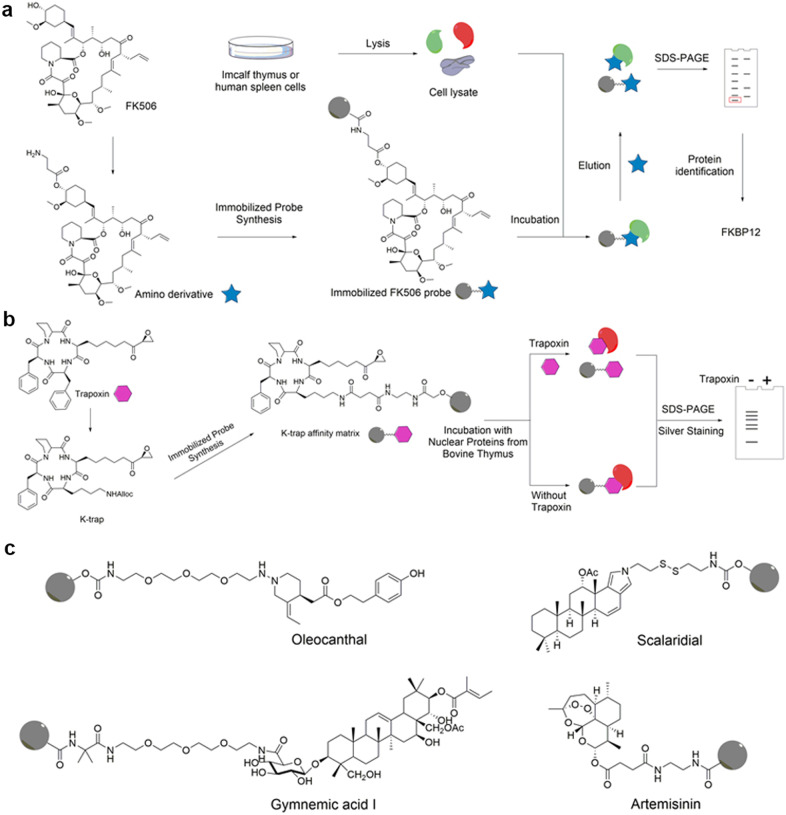
Workflow of target identification with immobilized probes of natural medicines. **a** Identification of FK506 protein targets with an immobilized FK506 probe. **b** Identification of trapoxin protein targets with K-trap. **c** Structures of other reported immobilized probes of natural medicines

For example, Schreiber et al.^46^ immobilized FK506 (tacrolimus), a natural immunosuppressant, to identify its protein targets in lysates obtained from calf thymus and human spleen cells. As shown in Fig. 2a, FK506 affinity matrices were prepared using an FK506 amino derivative. After complete incubation with cytosolic extracts of bovine thymus and human spleen, the matrix was competitively eluted with FK506, and a 14 K protein was enriched and identified. This led to the identification of a FK506-binding protein of 12 K (FKBP12), which functions as a protein folding chaperone for proteins containing proline residues. Another example is trapoxin, a microbially derived cyclotetrapeptide that inhibits histone deacetylation in vivo and causes mammalian cells to undergo cell cycle arrest.^51^ Because the epoxyketone side chain of trapoxin is indispensable for activity, Schreiber et al. chose to replace one of the phenylalanine residues of trapoxin’s cyclic core with a lysine that could then be covalently linked to a solid support. The matrix was incubated with nuclear proteins from bovine thymus, and the bound polypeptides were eluted by boiling the matrix in 1% SDS buffer. Six major polypeptides with apparent molecular sizes between 45 and 50 kDa were detected by SDS-PAGE and silver staining. In addition, the authors also employed trapoxin to competitively inhibit the binding between the polypeptides and the matrix to validate the specificity of the results (Fig. 2b). Other examples are illustrated in Fig. 2c.^52–55^

Although immobilized probes are easily synthesized and widely employed, one of their limitations, immobilization-induced steric hindrance, remains unsolved. The immobilization of the probes, which are always employed in CCCP, might influence the binding between the true targets and the reactive group, potentially leading to false-positive protein targets or the loss of potentially important protein targets. This could prove costly if an unsuitable protein target was selected for further evaluation.

### Activity-based probe

To overcome immobilization-induced activity impairment, activity-based probes (ABPs) were developed for target identification in chemical proteomics. In the design of such probes, the first factor to consider is the activity of the drug molecule;^31^ in other words, the incorporation of the reporter group and the linker should not influence the bioactivity of the active molecule. Therefore, the SAR of the molecule should be studied or consulted before the start of the synthesis, and the probe’s pharmacological activity should be determined. Unlike immobilized probes, ABPs can interact with proteins in the active proteome before enrichment and even pass through the cell membrane to bind target proteins in living cells, potentially reflecting the true drug–target interactions under physiological conditions in cells.

**Fig. 3:**
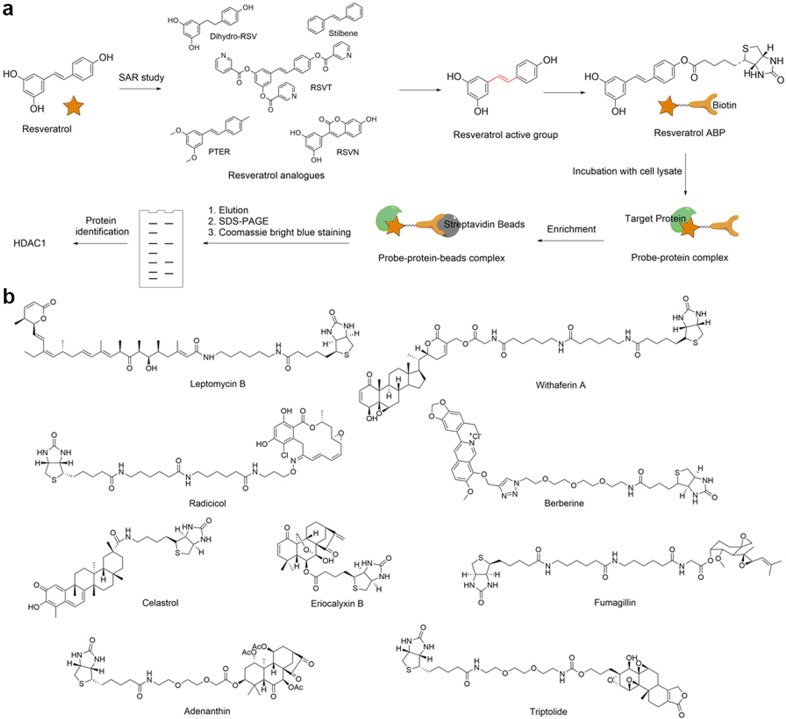
Schematic of target identification with activity-based probes of natural medicines. **a** Target identification of resveratrol with its activity-based probe. **b** Structures of some previously reported activity-based probes of natural medicines

However, non-immobilization raises an obvious question: How does one enrich or detect probes and target proteins is the probe is not immobilized? In the structure of ABP, a reporter group is present to overcome this problem (Fig. 1b). Among the diverse reporter groups, biotin is most frequently utilized due to its strong affinity with avidin, allowing its enrichment using either avidin or streptavidin beads. For instance, to elucidate the mechanism of resveratrol, a natural product exhibiting anticancer activity in mouse melanoma cells, our group synthesized a probe by connecting a biotin tag to resveratrol based on an SAR study and validated the probe activity with in vitro biochemical experiments (Fig. 3a). Subsequently, the probe was incubated with lysates of melanoma cells and then enriched with streptavidin beads. As a result, we identified histone deacetylase I (HDAC1) as the protein target of resveratrol in mouse melanoma cells and revealed an epigenetic regulation pathway of focal adhesion kinase.^41^ Other excellent examples are listed in Fig. 3b.^56–64^

In addition to the biotin tag, fluorescent tags are also widely used as the reporter group for target identification. Fluorescent-modified probes allow. the efficient and rapid detection of target proteins, but it cannot be enriched like biotin tags.^65^ However, in some cases, due to the large size of biotin, biotin can interfere with the original activity of the bioactive drug molecule. In addition, endogenous biotinylated proteins in the active proteome can interfere with identification by generating false-positive protein targets.^66,67^

### Click chemistry probe

With advancements in bioorthogonal chemical reactions, especially the development of the click reaction, the limitations of biotinylated probes have been largely alleviated. In fact, the incorporation of an orthogonally reactive group in the structure of natural products has been one of the most widely used strategies for target identification in the last decade. With the orthogonal reaction group, such probes can undergo bioorthogonal click reactions with their complements (e.g., azide to alkyne,^68^ strained alkene to tetrazine,^69^ tetrazine to cyclopropane,^70^ etc. and vice versa), thereby covalently connecting probes to affinity tags (biotin-azide, biotin-cyclopropane, etc.) or fluorescent tags (rhodamine-azide, FITC-azide, etc.) for subsequent enrichment and target identification (Fig. 1b). Due to their relatively small sizes, these orthogonally reactive groups have little or no influence on the intrinsic pharmacological activity of the natural products, and the probes can easily reach the cytoplasm to bind target proteins in situ before the click reaction and enrichment.

**Fig. 4:**
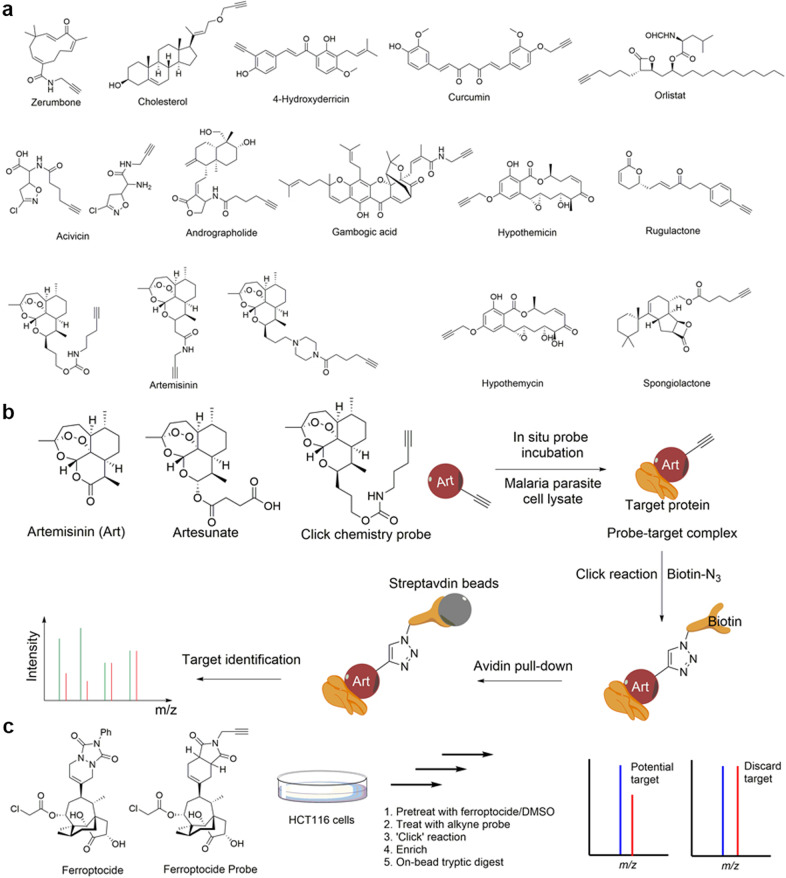
Schematic of target identification with click chemistry probes of natural medicines. **a** Structures of some reported click chemistry probes of natural medicines. **b** Identification of artemisinin protein targets with its click chemistry probe. **c** Identification of ferroptocide protein targets with its click chemistry probe

Many research groups, such as Tate’s group at Imperial College, Sieber’s group at the Technical University of Munich and Lin’s group at the National University of Singapore, have made great achievements in the target identification of natural products with click chemistry probes, including acivicin,^71^ curcumin,^67^ andrographolide,^72^ artemisinin,^73^ zerumbone^74^ and cholesterol.^75^ (Fig. 4a). Taking artemisinin as an example (Fig. 4b), Wang et al. utilized a click chemistry probe of artemisinin to identify its protein targets in *Plasmodium falciparum* and made two important findings: (i) heme, rather than free ferrous iron, is predominantly responsible for artemisinin activation; and (ii) artemisinin may kill the parasite through a promiscuous targeting mechanism. Because modifying artemisinin’s structure without influencing its activity is quite difficult, the authors synthesized a click chemistry probe derived from artesunate, an analog of artemisinin that also exhibits antimalarial activity. After activity validation, the probe was incubated with malaria parasites to fully bind the target proteins. Then, the target–probe complex was modified with a biotin tag through a click reaction and enriched with streptavidin beads. Finally, a total of 124 parasite proteins were identified, of which 33 proteins had previously been reported. Moreover, taking OAT (ornithine metabolism, arginine and proline metabolism) as a representative target, the activation mechanism of artemisinin was studied. It was observed that the probe itself did not bind to OAT, and its binding required the addition of hemin and was further enhanced in the presence of vitamin C, Na_2_S_2_O_4_ or glutathione, which reduce hemin to heme. By contrast, the addition of ferrous iron had no detectable effect on probe-OAT binding, revealing that heme, rather than free ferrous iron, has a predominant role in artemisinin activation.

Another example is ferroptocide,^76^ a small molecule chemically derived from the diterpene natural product pleuromutilin that rapidly and robustly induces ferroptotic death of cancer cells. After biological evaluation, Llabani et al. synthesized a click chemistry probe of ferroptocide to identify the protein targets in HCT 116 cells (Fig. 4c). The cells were pretreated with 20 μM ferroptocide or DMSO for 60 min and then incubated with 20 μM ferroptocide probe for 30 min; a click reaction with biotin-azide and enrichment with streptavidin magnetic beads followed. On-bead trypsin digestion coupled to LC/LC–MS/MS analysis provided a list of over 300 targets. With subsequent CRISPR knockout studies, the authors found that ferroptocide is an inhibitor of thioredoxin, a key component of the antioxidant system in the cell, and positively modulates the immune system in a murine model of breast cancer.

### Photoaffinity probe

All the click chemistry probes mentioned above possess active groups that can covalently modify the amino acid residues in target proteins, leading to steady binding between the probes and the targets during the click reaction and enrichment. However, some natural products, such as resveratrol, interact with their protein targets through noncovalent secondary bonds, including hydrogen bonds, ionic bonds and hydrophobic interactions. For these bioactive molecules, simple click chemistry probes are unsuitable for target identification because the binding between the active molecule and the target protein can be disrupted during the click and enrich processes due to their noncovalent interactions. For such cases, the photoaffinity labeling technique (PAL) was developed.^77–79^

Photoaffinity probes generally consist of a click chemistry probe skeleton for target binding and enrichment and a photoaffinity group for fixing the binding between the probe and the targets (Fig. 1b). After incubation with the active proteome, the photoaffinity probe generates highly active free radical intermediates to covalently bind the target protein under certain wavelengths of light, and this is followed by click chemistry and target enrichment. Frequently used photoaffinity groups include benzophenone, aryl azide, and diazirine, and of these, benzophenone is the most widely applied due to its stability and ease of synthesis.^80,81^ In recent years, diazirine-based photoaffinity probe synthesis has attracted much interest due to its small size and high efficiency. Unfortunately, photoaffinity groups can intrinsically bind some nonspecific proteins (e.g., diazirine to voltage-dependent anion channels),^82,83^ which may affect the accuracy of the results.

**Fig. 5:**
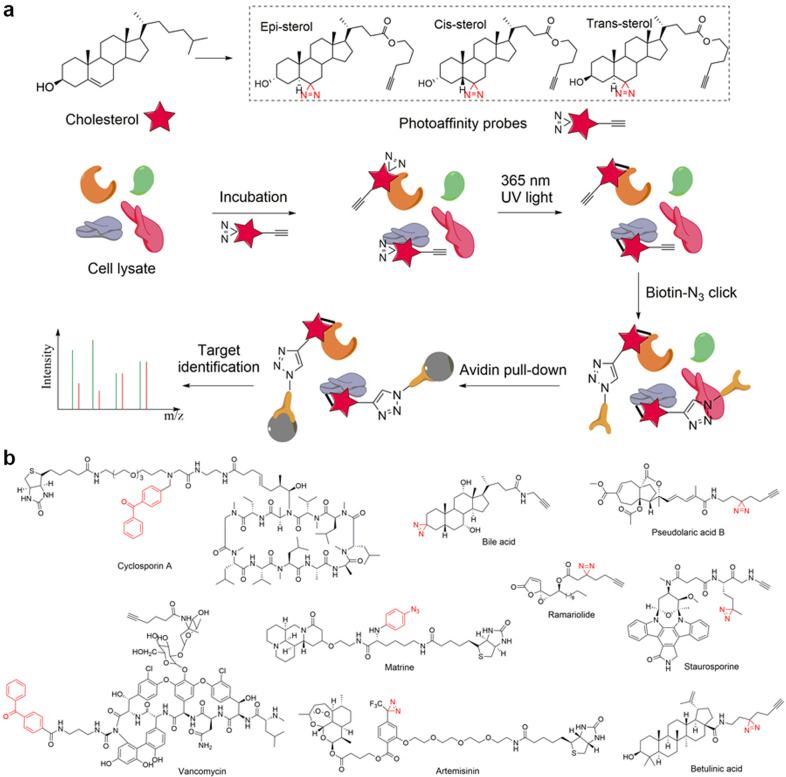
Schematic of target identification with photoaffinity probes of natural medicines. **a** Cholesterol target identification with its photoaffinity probe. **b** Structures of some reported photoaffinity probes of natural medicines. The photoaffinity groups are indicated in red

Cravatt’s group has utilized photoaffinity probes directly in living mammalian cells to globally map the binding proteins of cholesterol, an essential structural component of cellular membranes that serves as a precursor of several classes of signaling molecules.^84^ Based on cholesterol’s structure, they first designed and synthesized a set of sterol probes (Fig. 5a), each of which contained a photoactivatable diazirine group at the 6 position of the steroid core, which is a modification that has previously been shown to minimally perturb the biophysical properties of cholesterol. Then, living human cells were incubated with the probes and irradiated with 365 nm UV light to covalently cross-link the probe with the targets. After biotin modification through a click reaction and enrichment with streptavidin beads, the target proteins were identified with a quantitative proteomics approach. Over 250 cholesterol-binding proteins, including receptors, channels and enzymes involved in many established and previously unreported interactions, were identified. Other examples of photoaffinity probes are illustrated in Fig. 5b.^85–93^

### Nonlabeling approach

**Fig. 6:**
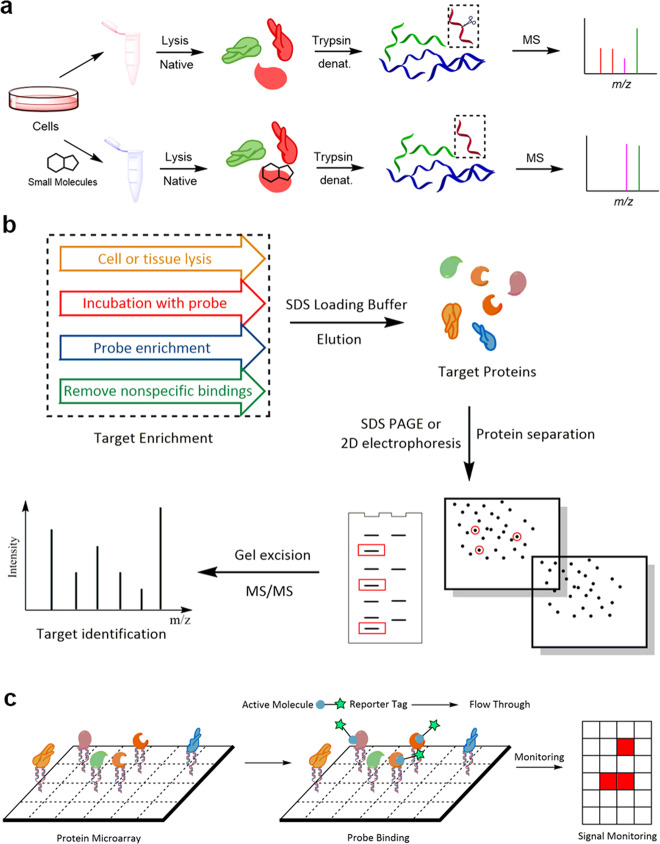
**a** Schematic of target identification of ATP, PEP and l-Phe with DARTS. **b** Workflow of target fishing and MS identification. **c** Workflow of chemical proteomics combined with protein microarray

In all the types of probes described above, the addition of an exogenous group could interfere with the pharmacological activity of the natural product. Moreover, for some natural products, their structures have no active sites suitable for modification, thereby limiting the application of these probe-based methods. For this reason, some nonlabeling chemical proteomics approaches, such as drug affinity responsive target stability (DARTS),^94,95^ stability of proteins from rates of oxidation (SPROX),^96,97^ cellular thermal shift assay (CETSA)^98,99^ and thermal proteome profiling (TPP),^100^ were developed for target identification. DARTS identifies targets by detecting enzymolysis changes based on the fact that the binding of the drug molecule stabilizes target proteins to trypsin-induced hydrolysis. For instance, Piazza et al.^101^ utilized a DARTS-based approach to identify the protein targets of three metabolites: adenosine 5′-triphosphate (ATP), l-phenylalanine (l-Phe) and phosphoenolpyruvate (PEP). In the study (Fig. 6a), proteomes were extracted under conditions that preserve the structures of native proteins, and the extracts were exposed to the small molecule of interest. Samples were subjected to limited proteolysis with the broad-specificity protease proteinase K to generate structure-specific protein fragments. The fragments were then digested with the sequence-specific protease trypsin to generate peptide mixtures amenable to bottom-up proteomic analysis. The peptides were analyzed with MS, and the targets were identified by comparing the peptides in the presence and absence of the small molecule. A total of 231 targets were observed for ATP, and 129 and 41 protein targets were identified for PEP and l-Phe, respectively.

Unlike DARTS, SPROX detects the oxidation level of methionine in proteins to identify targets due to changes in antioxidant ability following the binding of the molecule.^96^ CETSA covers a wider range of applications than DARTS and SPROX, such as target identification in living cells, cell lysates and tissues. It is based on the thermodynamic stability alterations induced by the drug molecule’s binding. To overcome the challenges of low sensitivity and throughput in CETSA, TPP was developed.^100^ It is derived from CETSA but also allows the identification of off-targets and biomarkers. For these nonlabeling methods, the natural products need not be modified or attached to exogenous groups, allowing complete retention of their intrinsic bioactivities. Although nonlabeling chemical proteomics approaches have been widely applied in the target identification of natural products and medicines,^97,102^ they suffer from a few drawbacks, such as the tedious condition groping process and insufficient target sensitivity against nonlabeling molecules.

## Target identification

After probe synthesis and subsequent target enrichment, the target proteins are identified with proteomic analysis by mass spectrometry, which is a valuable tool.^103,104^ In the early days of research, mass spectrometry was applied to identify specific bands in SDS electrophoretic gels or spots in two-dimensional electrophoretic gels.^105,106^ Because the targets are confirmed by comparing the gray values of proteins in different groups (probe vs control), this method may lead to low-abundance target loss and nonspecific results. To overcome these challenges, quantitative proteomics approaches for measuring abundance changes of many proteins in multiple samples have been developed.^107–109^ In addition, with the development of chip technology, protein microarrays have also been employed for target identification in chemical proteomics approaches.^110,111^ In this section, we will describe several different protein identification methods in detail, including their workflows, features and practical applications.

### Gel separation and band identification

Briefly, the target proteins in the active proteome are enriched with molecule-derived probes and subsequently separated through SDS-PAGE or two-dimensional electrophoresis. Coomassie brilliant blue staining or silver staining can be used to visualize the separated proteins. Then, the gels containing the enriched proteins from different groups (always probe vs control) were aligned to identify the distinct bands or spots, followed by gel excision and in-gel digestion. Finally, the target proteins were identified by identifying the postdigested peptides with mass spectrometry (Fig. 6b). In the example mentioned above, our group identified resveratrol’s targets in mouse melanoma cells with this method (Fig. 3a). After probe synthesis and target enrichment, the binding proteins were eluted with SDS loading buffer. Then, the targets were separated by SDS-PAGE, followed by Coomassie brilliant blue staining, and we discovered two distinct bands compared with the control lane (DMSO). The two bands were excised and identified as acetyl-CoA acetyltransferase 1 (ACAT1) and HDAC1. With in vivo and in vitro experiments, we confirmed that resveratrol inhibits focal adhesion kinase (FAK) expression by interacting with HDAC1.^41^ Although the method has been widely used in protein identification,^112–114^ it has two disadvantages: (i) some low-abundance but vital target proteins are still invisible after Coomassie brilliant blue staining or even silver staining, resulting in target loss; and (ii) some distinct bands or spots, especially bands, contain more than one protein, so the nonspecific binding in these bands or spots may also be identified.

### Quantitative proteomics

To overcome the deficiencies in-gel separation and band identification, quantitative methods have been incorporated into chemical proteomics.^40,115–118^ By comparing the relative abundance of proteins between different samples or against appropriate negative controls, proteins with a higher abundance ratio (>1.5 or 2, different values have been used) than a threshold are identified as specific targets, thus avoiding nonspecific binders. Due to the precision of the method, low-abundance targets can also be identified.^119^ Quantitative proteomics approaches mainly include stable isotope labeling by amino acids in cell culture (SILAC), chemical labeling approaches and label-free approaches.

**Fig. 7:**
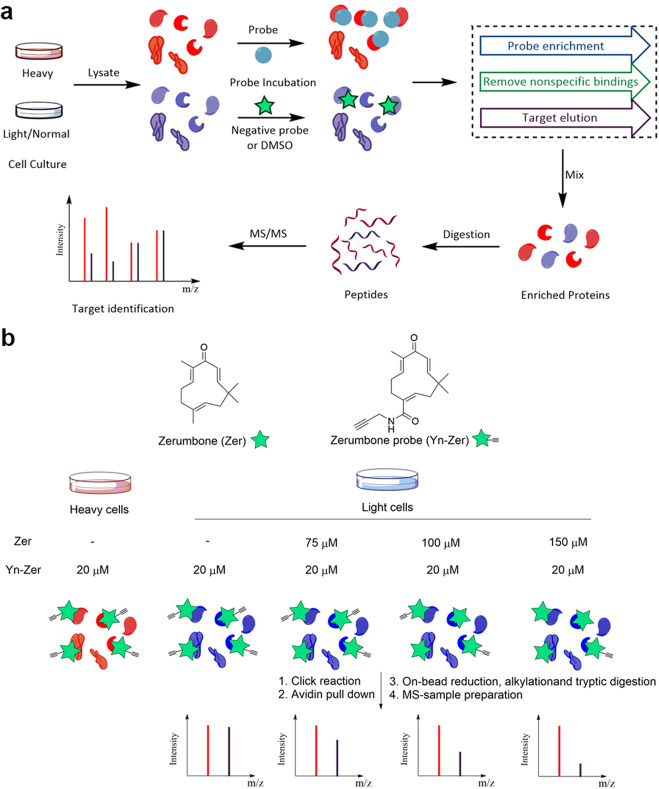
Schematic of protein identification with SILAC. **a** General workflow of chemical proteomics combined with SILAC. **b** Target identification of zerumbone through a chemical proteomics approach coupled with SILAC

SILAC has become the most frequently used quantitative proteomics approach in target identification since its development in 2002.^120^ It uses isotopically labeled amino acids to stably label proteins during cell culture and determines the relative quantity of peptides by comparing the molecular weight shifts after MS. As the initial step of a typical SILAC experiment, two parallel cell populations are cultured in different media (one contains normal amino acids and another contains isotopically labeled amino acids, namely, “heavy” amino acids^121^) resulting in molecular weight differences in the newly synthesized proteins between the two populations after several generations of cell culture (Fig. 7a). Next, the synthesized probe and negative control (usually DMSO) are incubated with the heavy cells and normal cells, respectively. After “target fishing”, the enriched proteins from the two groups are pooled for subsequent identification to avoid measurement error. According to the shift in the molecular weight of the proteins from the two groups, the specific target proteins can be easily identified by comparing the relative protein abundances between the two groups.

To date, many studies have applied chemical proteomics approaches along with SILAC to identify protein targets of various natural products as well as natural medicines. For instance, Kalesh et al.^74^ applied a “spike-in” SILAC method to identify the cellular targets of zerumbone (Zer), a phytochemical with diverse biological activities ranging from anti-inflammatory to anticancer properties. As shown in Fig. 7b, the authors initially synthesized a click chemistry probe of zerumbone (Yn-Zer), and the probe’s activity was confirmed to be similar to that of zerumbone. In parallel, lysates of HeLa cells labeled with ^15^N_4_^13^C_6_-arginine and ^15^N_2_^13^C_6_-lysine (heavy cells) were treated with 20 μM Yn-Zer, whereas lysates from cells cultured in normal medium (light cells) were treated with 20 μM Yn-Zer, 20 μM Yn-Zer combined with 75 μM Zer, 20 μM Yn-Zer combined with 100 μM Zer, and 20 μM Yn-Zer combined with 150 μM Zer. The addition of Zer competitively inhibited the binding between the target proteins and Yn-Zer. With SILAC, the relative amounts of the fished proteins (compared to the heavy group) were determined, and proteins with lower relative amounts with increasing Zer concentration were identified as specific targets. Finally, a total of 62 proteins that are involved in vital biological processes showed statistically significant enrichments, with many of these proteins having key roles in regulating apoptosis and cell survival.

**Fig. 8:**
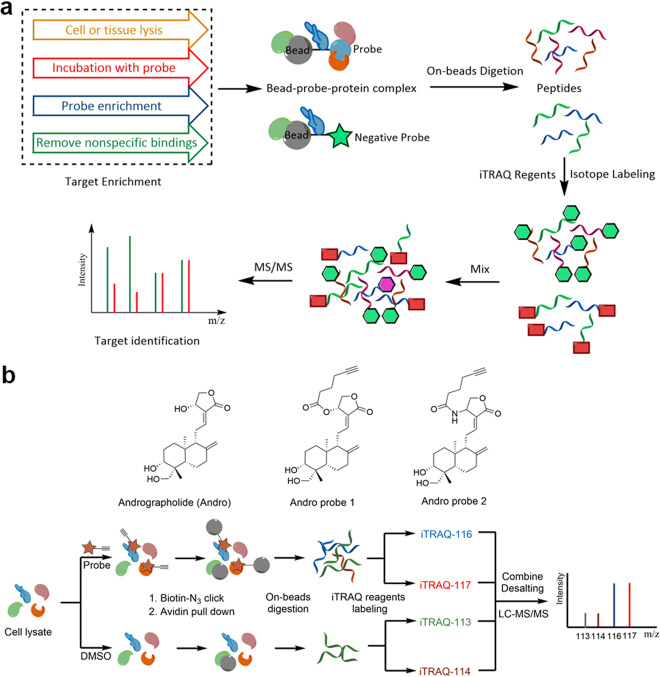
Schematic of protein identification with iTRAQ. **a** Workflow of chemical proteomics combined with iTRAQ. **b** Andrographolide target identification with chemical proteomics combined with iTRAQ

Compared with SILAC, isobaric tags for relative and absolute quantification (iTRAQ), a typical chemical labeling approach, can be used to perform stable isotope labeling of peptides digested from proteins and utilizes labeling reagents to quantify reporter ions fragmented by MS/MS,^119^ affording many advantages.^122,123^ For example, in some applications, such as identifying targets in natural microbial communities or primary tissue samples, SILAC is not suitable due to its complicated labeling process during cell culture, whereas iTRAQ is postapplicative.^124^ Moreover, in a SILAC experiment, at most three samples can be determined at one time, whereas iTRAQ can simultaneously analyze up to eight samples.^125^ The general workflow for target identification with iTRAQ is illustrated in Fig. 8a, and it only differs from SILAC in the labeling process. In iTRAQ, the peptides digested from proteins of different groups are incubated with different iTRAQ regents for isotope labeling. Due to the mass difference of reporter ions in different iTRAQ reagents, the relative protein abundance in different groups can be calculated. iTRAQ has also been widely applied in the identification of targets of natural medicines.^126,127^

For example, Wang et al.^72^ utilized a clickable activity-based probe derived from andrographolide, a natural product with known anti-inflammatory and anticancer effects, to enrich protein targets in live cancer cells (Fig. 8b). In the assay, the cells were first incubated with the clickable probe or with DMSO as a negative control. Following sequential probe binding, biotin modification through a click reaction, avidin pull-down and thorough washing, the target proteins were digested with trypsin. The resulting peptides were labeled with their respective iTRAQ regents (control group with 113 and 114, whereas probe group with 116 and 117) and pooled for further identification and quantification via LC–MS/MS. The results were also validated through cell migration and invasion assays, revealing that andrographolide has a potential novel application as a tumor metastasis inhibitor.

For the label-free quantitative proteomics approach, protein abundance is calculated by detecting the MS signal densities of peptides digested from the specific protein or the number of MS/MS signals corresponding to peptides and proteins.^128^ Due to the missing labeling process, this method is much simpler and more cost-efficient.^129^ However, it has disadvantages in accuracy and throughput, especially in some promiscuous cases, such as samples with heavy backgrounds, compared to quantitative labeling proteomics approaches.^130,131^ Moreover, in the label-free approach, only one MS run can be done per sample, so samples of different groups have to be examined in separate MS runs, which might lead to operating error, leading to reduced accuracy in the results.

### Protein microarray

In addition to MS-based approaches to identify the protein targets of drug molecules of interest, protein microarrays are another approach that has been utilized frequently. The main function of protein microarray technology is investigating the functional properties of immobilized proteins, the interaction of proteins, and their enzyme activity, and it has also been utilized to study the interactions between proteins and natural products or medicines for decades.^132,133^ Combined with chemical proteomics, this method can also be applied for target identification, providing a platform for analyzing the interactions between small molecules and thousands of proteins.^134^ In a typical protein microarray approach (Fig. 6c), diverse proteins are first immobilized on a miniature high-density array, followed by the labeling of the molecule to be tested with an affinity tag, such as biotin, a fluorophore, a photoaffinity group or a radioactive isotope, to allow the molecule-linked proteins to be easily traced. Notably, this method is high-throughput, allowing the identification of target and off-target proteins in the whole proteome on the microarray in a single run.^135,136^ However, it also possesses certain disadvantages. For example, active molecules need to be modified with tags that might influence their intrinsic activities. Along with the development of mass spectrometry techniques, protein microarrays are always combined with mass spectrometry to overcome modification-induced activity alterations.^137^

## Summary and outlook

In the development of new natural medicines, target identification facilitates the determination of the MOA and side effects, accelerating this process from discovery to market. Along with the development of chemical biology and proteomics, the chemical proteomics approach has become a popular method in target identification of small active molecules, especially natural products, providing an important theoretical basis for novel natural medicine research and development. In most cases, natural products need to be modified with reporter tags to facilitate enrichment or detection, which might influence their intrinsic pharmacological activities, thereby leading to a biased target result. Moreover, some nonlabeling chemical proteomics approaches for target identification are not well qualified in promiscuous cases, and their low accuracy and throughput limit their broad application. Therefore, developing a highly accurate nonlabeling chemical proteomics approach with high-throughput is imperative. Although some studies have simultaneously applied two or more different strategies for target identification to avoid nonspecific binding and narrow target collection and obtained a more accurate target list, the MOA predicted from the targets must be validated by biochemical methods. Collectively, the use of chemical proteomics will continue to be a key tool to drive the discovery of new therapeutic compounds of natural origin.
